# Lappaol F, an anticancer agent, inhibits YAP via transcriptional and post-translational regulation

**DOI:** 10.1080/13880209.2021.1923759

**Published:** 2021-05-19

**Authors:** Xiao Li, Yi-Ying Lin, Jia-Yi Tan, Kang-Lun Liu, Xiao-Ling Shen, Ying-Jie Hu, Rui-Yi Yang

**Affiliations:** Science and Technology Innovation Center, Guangzhou University of Chinese Medicine, Guangzhou, China

**Keywords:** Yes-associated protein, 14-3-3σ, tumour suppression, cell proliferation, apoptosis, colon xenografts

## Abstract

**Context:**

Lappaol F (LAF), a natural lignan from *Arctium lappa* Linné (Asteraceae), inhibits tumour cell growth by inducing cell cycle arrest. However, its underlying anticancer mechanism remains unclear.

**Objective:**

The effects of LAF on the Hippo-Yes-associated protein (YAP) signalling pathway, which plays an important role in cancer progression, were explored in this study.

**Materials and methods:**

Cervical (HeLa), colorectal (SW480), breast (MDA-MB-231) and prostate (PC3) cancer cell lines were treated with LAF at different concentrations and different durations. BALB/c nude mice bearing colon xenografts were intravenously injected with vehicle, LAF (10 or 20 mg/kg) or paclitaxel (10 mg/kg) for 15 days. The expression and nuclear localisation of YAP were analysed using transcriptome sequencing, quantitative PCR, western blotting and immunofluorescence.

**Results:**

LAF suppressed the proliferation of HeLa, MDA-MB-231, SW480 and PC3 cells (IC_50_ values of 41.5, 26.0, 45.3 and 42.9 μmol/L, respectively, at 72 h), and this was accompanied by significant downregulation in the expression of YAP and its downstream target genes at both the mRNA and protein levels. The expression of 14-3-3σ, a protein that causes YAP cytoplasmic retention and degradation, was remarkably increased, resulting in a decrease in YAP nuclear localisation. Knockdown of 14-3-3σ with small interfering RNA partially blocked LAF-induced YAP inhibition and anti-proliferation effects. In colon xenografts, treatment with LAF led to reduced YAP expression, increased tumour cell apoptosis and tumour growth inhibition.

**Conclusion:**

LAF was shown to be an inhibitor of YAP. It exerts anticancer activity by inhibiting YAP at the transcriptional and post-translational levels.

## Introduction

Cancer is a serious disease that threatens human health and is currently a leading cause of death worldwide. According to recent research, an estimated 18.1 million new cancer cases were diagnosed, and 9.6 million cancer deaths occurred worldwide in 2018 (Bray et al. 2018). In addition to surgery and radiotherapy, chemotherapy is the main treatment for cancer. In the 1950s, the National Cancer Institute (NCI) launched a research program for screening of anticancer drugs from plant extracts. For decades, the discovery and application of natural anticancer drugs, such as paclitaxel (isolated from *Taxus brevifolia* Nutt., Taxaceae), vinca alkaloids (vinblastine and vincristine, isolated from *Catharanthus roseus* [Linn.] G. Don, Apocynaceae) and camptothecin (isolated from *Camptotheca acuminata* Decne., Nyssaceae), have greatly reduced the number of cancer-associated deaths and improved the cure rate (Sirikantaramas et al. 2007; Bernabeu et al. 2017; Martino et al. 2018). Furthermore, derivatives designed and synthesised based on the structures of plant-derived drugs have also been widely used in the treatment of human cancers (Meng et al. 2016; Amin et al. 2018). It is estimated that more than 50% of anticancer drugs used in clinical settings are natural products or their derivatives (Amin et al. 2009). Although existing chemotherapeutic drugs are very useful in controlling the progression of cancer, adverse reactions and drug resistance often lead to treatment failure. Therefore, there is an urgent need to discover and develop safer and more effective anticancer agents.

*Arctium lappa* L. (Asteraceae), commonly known as burdock, is often used as an anticancer, anti-inflammatory, antiviral, and antidiabetic agent in traditional Chinese medicine (Chan et al. 2011). Lappaol F (LAF) is a lignanoid compound isolated from its seeds. It has been reported that LAF has antiaging activity, inhibits lipopolysaccharide-induced nitric oxide production and reverses multidrug resistance (Park et al. 2007; Su et al. 2015; Su & Wink 2015). Our previous study showed that LAF had a strong killing effect on a variety of cancer cell lines through cell cycle arrest, with minimal cytotoxicity towards normal cell lines. Moreover, LAF significantly suppressed the growth of cervical cancer xenografts in a nude mouse model (Sun et al. 2014). However, the targets responsible for the antitumour activity of LAF are currently not well understood.

Yes-associated protein (YAP) is a nuclear transcriptional activator that activates the transcription of many oncogenes. Thus, YAP plays a crucial role in cancer progression and is expected to be a promising anticancer drug target (Wu & Yang 2018). In this study, we investigated the effect of LAF in inhibiting the expression and function of YAP. We hypothesised that this may be the underlying mechanism of LAF in cancer treatment.

## Materials and methods

### Chemicals and reagents

LAF (C_40_H_42_O_12_, M = 714) was extracted from the seeds of *Arctium lappa*, as previously described (Sun et al. 2014); a yield and purity of 0.03% and 98.0%, respectively, were determined by high-performance liquid chromatography. Verteporfin (VP) and recombinant human epidermal growth factor (EGF) were purchased from Selleck Chemicals (Houston, TX, USA) and PeproTech (Rocky Hill, NJ, USA), respectively. LAF and VP were dissolved in dimethyl sulfoxide (DMSO) as stocks and diluted with cell growth medium to obtain the working concentration before use. EGF was dissolved and diluted with cell growth medium. Sulforhodamine B (SRB) was obtained from Sigma-Aldrich Co. (St. Louis, MO, USA). Control small interfering RNA (siRNA), 14-3-3σ siRNA and siRNA transfection reagent were obtained from Santa Cruz Biotechnology (Dallas, TX, USA). Paclitaxel injection was purchased from Corden Pharma Latina S.P.A (Sermoneta, Latina, Italy). An *in situ* cell death detection kit (Fluorescein) was purchased from Roche (Basel, Switzerland). The Annexin V-FITC/PI apoptosis kit was purchased from Multi Sciences (Hangzhou, China).

### Cell culture and proliferation assay

HeLa (cervix), MDA-MB-231 (breast), SW480 (colon) and PC3 (prostate) human cancer cell lines were obtained from the American Type Culture Collection (Manassas, VA, USA). HeLa, SW480 and PC3 cells were grown in Roswell Park Memorial Institute (RPMI)-1640 medium, and MDA-MB-231 cells were grown in Dulbecco’s Modified Eagle’s medium. All media were supplemented with 10% foetal bovine serum (FBS), and cells were incubated at 37 °C and cultured in a 5% CO_2_ atmosphere. For proliferation assays, cells were plated in 96-well plates (7.5 × 10^3^ cells per well). After treatment with LAF, an SRB assay was performed to assess cell proliferation.

### Flow cytometry for apoptosis analysis

HeLa, MDA-MB-231, SW480 and PC3 cells were seeded in 6-well plates (3.75 × 10^5^ cells per well) and grown overnight. LAF was added to the wells and the cells were cultured for 48 h. Both viable and dead (floating) cells were collected and resuspended using an Annexin V-FITC/PI apoptosis kit according to the manufacturer’s instructions. The analysis was performed using a NovoCyte 2060 R flow cytometer (ACEA Bioscience, Inc., San Diego, CA, USA).

### Transcriptome profiling

SW480 cells were treated with 50 μmol/L LAF for 12, 24 or 36 h, and total RNA was isolated using TRIzol reagent. Transcriptome sequencing was performed by BioMarker Technologies (Beijing, China). Differentially expressed genes (DEGs) with a fold change ≥ 2 and false discovery rate (FDR) < 0.01 were identified. The DEGs were subjected to functional enrichment analysis based on the Kyoto Encyclopaedia of Genes and Genomes (KEGG) pathways.

### Quantitative RT-PCR

Total RNA was isolated from cells using TRIzol. Reverse transcription was conducted using the PrimeScript^TM^ RT reagent kit (TaKaRa, Shanghai, China) according to the manufacturer’s instructions. mRNA levels of YAP and 14-3-3σ were analysed by real-time fluorescent quantitative PCR with TB Green Premix Ex Taq^TM^ II (Tli RNaseH Plus, TaKaRa) using an ABI 7500 system (Applied Biosystems, Foster City, CA). The gene encoding glyceraldehyde-3-phosphate dehydrogenase (GAPDH) was used as an internal reference. The primers were designed as follows: YAP, forward 5′-TAGCCCTGCGTAGCCAGTTA-3′ and reverse 5′-TCATGCTTAGTCCACTGTCTGT-3′; 14-3-3σ, forward 5′-TGACGACAAGAAGCGCATCAT-3′ and reverse 5′-GTAGTGGAAGACGGAAAAGTTCA-3′; GAPDH, forward 5′-GGAGCGAGATCCCTCCAAAAT-3′ and reverse 5′-GGCTGTTGTCATACTTCTCATGG-3′.

### Xenograft experiment

The animal experiments were approved by the Animal Ethics Committee of Guangzhou University of Chinese Medicine (No. 20181101002) and were performed in accordance with the stipulated guidelines. Female BALB/c nude mice (4-5 week-old) were purchased from the Nanjing Biomedical Research Institute of Nanjing University. To establish a xenograft model, 4 × 10^6^ SW480 cells in 100 μL of serum-free medium were subcutaneously injected into the left and right back regions of the nude mice. Five days later, the tumour-bearing mice were randomised into 4 groups (8 mice in each group): vehicle, LAF at 10 mg/kg, LAF at 20 mg/kg and paclitaxel at 10 mg/kg. LAF was dissolved in a mixed solution consisting of equivalent quantities of ethanol, Tween^®^-80 and Cremophor EL, then diluted with saline to form an injectable solution containing 0.5% LAF, 2.8% ethanol, 2.8% Tween^®^-80 and 2.8% Cremophor EL. This solution was intravenously administered to mice for 15 consecutive days. Mice in the vehicle group were injected with an equivalent volume of the solvent. Mice in the paclitaxel group were treated with paclitaxel injection (6 mg paclitaxel, 527 mg Cremophor EL and 49.7% ethanol per mL, diluted with saline before use) every other day (Zhu et al. 2014). Tumour volumes were recorded every four days and calculated according to the following formula: length × width^2^ × 0.5. At the end of the experiment, tumour xenografts were excised, weighed and preserved at −80 °C or with 4% polyformaldehyde for subsequent analyses.

### Terminal deoxynucleotidyl transferase–mediated dUTP nick end labelling (TUNEL) analysis

Apoptosis in tumour xenografts was detected using an *in situ* cell death detection kit based on the TUNEL technology. Briefly, paraffin-embedded tissue sections were dewaxed by washing in xylene and rehydrated through a graded series of ethanol and ultrapure water. After 15 min of pre-treatment with proteinase K working solution (20 μg/mL in 10 mmol/L Tris-HCl, pH 7.4) at 37 °C, the slices were incubated in TUNEL reaction mixture for 1 h at 37 °C in a humidified atmosphere in the dark. The nuclei were stained with 5 μg/mL 4,6-diamino-2-phenyl indole (DAPI) solution, and confocal microscopy (Zeiss LSM 510, Germany) was used to observe apoptosis.

### siRNA transfection and LAF treatment

Cells were plated in a 35 mm glass-bottom dish (2 × 10^6^ cells per dish) and left overnight. For each transfection, 1 μg of siRNA duplex (14-3-3σ or control) mixed with 6 μL siRNA transfection reagent was incubated with cells according to the manufacturer’s protocol. Twenty-four hours after transfection, 50 μmol/L LAF was added, and the cells were incubated for 24 h. The expression levels of 14-3-3σ and YAP were validated by western blotting and immunofluorescence assays.

### Western blotting and immunofluorescence analysis

To extract total protein, cells and tissues were dissolved in radioimmunoprecipitation assay lysis buffer containing protease inhibitors (Thermo Scientific #78425, Rockford, IL, USA; MedChemExpress LLC #HY-K0023, Monmouth Junction, NJ, USA) and cleared by centrifugation. Western blotting was performed as previously described (Song et al. 2019) using antibodies targeting the following proteins: YAP (Abcam, ab52771), p-YAP (Ser127) (Cell Signalling Technology [CST], #13008), 14-3-3σ (Santa Cruz, sc-100368), BIRC5 (CST, #2808), Bcl-2 (CST, #4223), c-Myc (CST, #13987), β-actin (Beijing ZhongShan JinQiao Biotechnology Co. LTD, #TA-09), goat anti-rabbit IgG-HRP (Abcam, ab6721) or goat anti-mouse IgG-HRP (Abcam, ab97040). For immunofluorescence assays, cells were cultured in a 35-mm glass-bottom dish (2 × 10^6^ cells per dish). Following drug intervention, the cells were fixed with 4% formaldehyde for 20 min and permeabilised using 0.5% Triton X-100 on ice for 10 min. After blocking with 2% bovine serum albumin for 1 h, the cells were incubated overnight at 4 °C with antibodies against YAP (Abcam, ab52771) or 14-3-3σ (Abcam, ab14123) diluted 1:100 in phosphate-buffered saline. Fluorescence-conjugated secondary antibody (Abcam, ab150077 or ab150115) was applied for 1 h at 25 °C, followed by incubation in DAPI solution for 5 min. The cells were immediately visualised using a confocal microscope.

### Statistical analysis

SPSS software, version 24.0 (SPSS, Inc., Chicago, IL, USA) was used for statistical analysis. All data are expressed as the mean ± standard deviation (SD). Intergroup and intragroup differences were calculated using one-way analysis of variance (ANOVA) with *post hoc* Tukey test. Statistical significance was set at *p* < 0.05. Each experiment was repeated at least three times.

## Results

### LAF suppressed proliferation and promoted apoptosis in various cancer cell types

**Figure 1.**
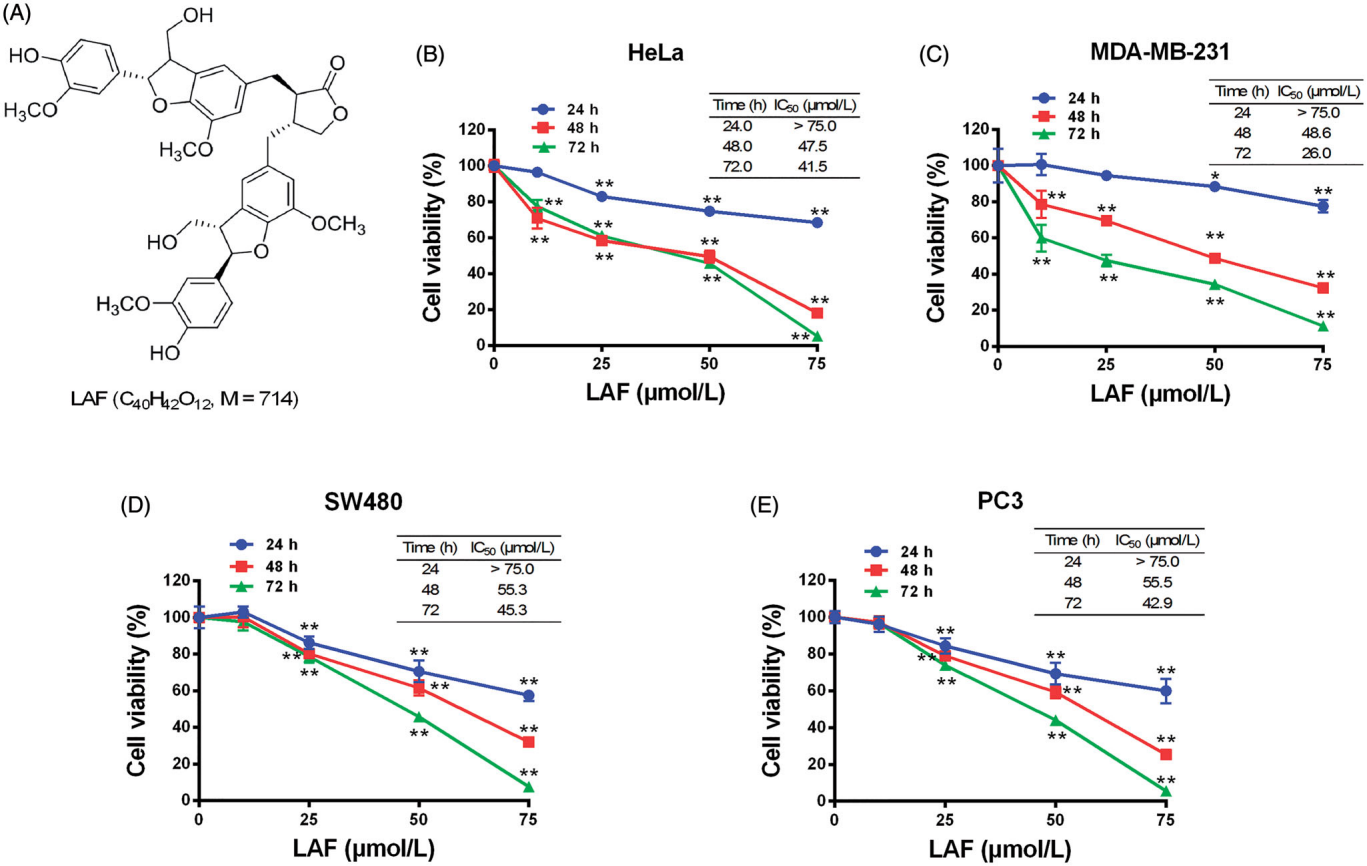
Lappaol F (LAF) inhibited viability of human cancer cells. (A) Structure of LAF. (B-E). Human cancer cells (HeLa, MDA-MB-231, SW480 and PC3) were treated with LAF (0, 10, 25, 50 or 75 µmol/L) for 24, 48 or 72 h. Cell viability was assessed by sulforhodamine B assay. All data are expressed as the mean ± SD (*n* = 5). **p < 0.01, significantly different from the control without LAF treatment.IC_50_: half inhibitory concentration.

**Figure 2.**
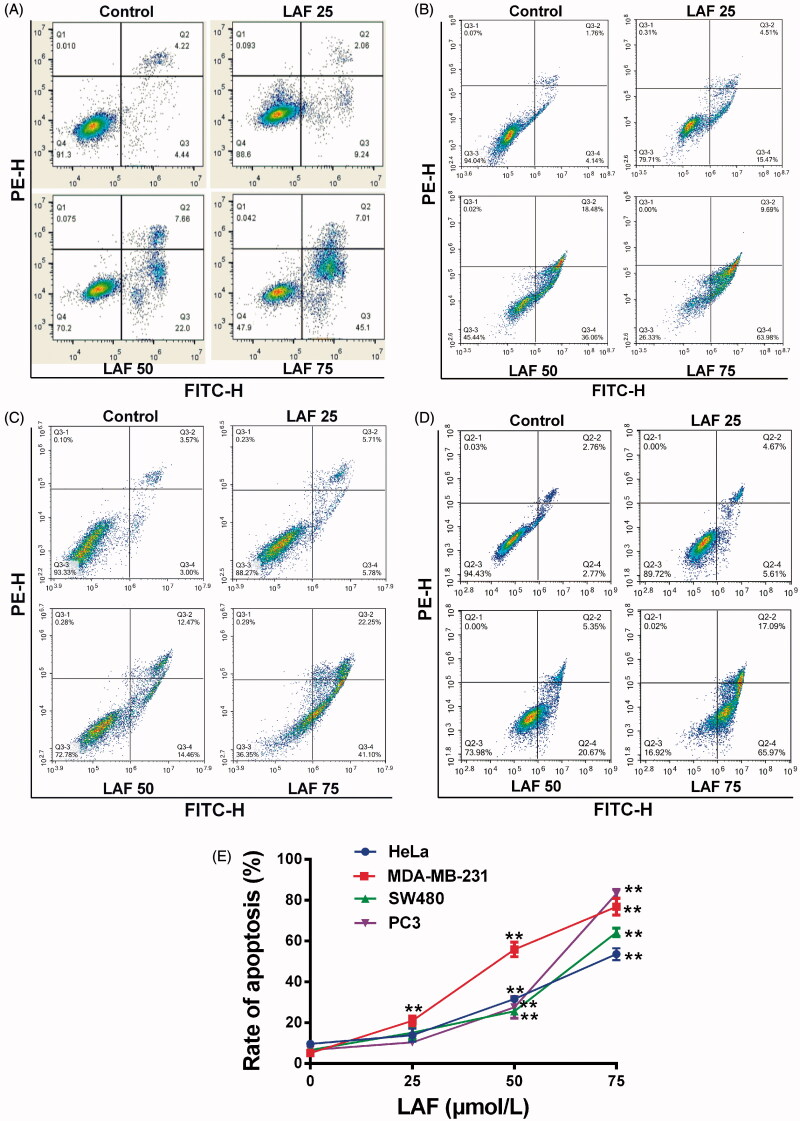
Lappaol F (LAF) promoted apoptosis in cancer cells. LAF (0, 25, 50 or 75 µmol/L) was incubated with cancer cells for 48 h. The rate of apoptosis was analysed by flow cytometry after annexin V-FITC/PI staining. (A) Representative plots of HeLa cells. (B) Representative plots of MDA-MB-231 cells. (C) Representative plots of SW480 cells. (D) Representative plots of PC3 cells. (E) Quantification of apoptosis (including early and late apoptotic cells). All data are expressed as the mean ± SD (*n* = 3). **p < 0.01, significantly different from the control without LAF treatment.

In this study, the effects of LAF on human cancer cell proliferation were assessed. LAF inhibited the growth of HeLa, MDA-MB-231, SW480 and PC3 cells. The viability curve and half-inhibitory concentration (IC_50_) at different times are depicted in Figure 1. In addition, LAF significantly promoted apoptosis in the aforementioned cancer cell lines (Figure 2).

### LAF downregulated YAP while upregulated 14-3-3σ at mRNA levels

**Figure 3.**
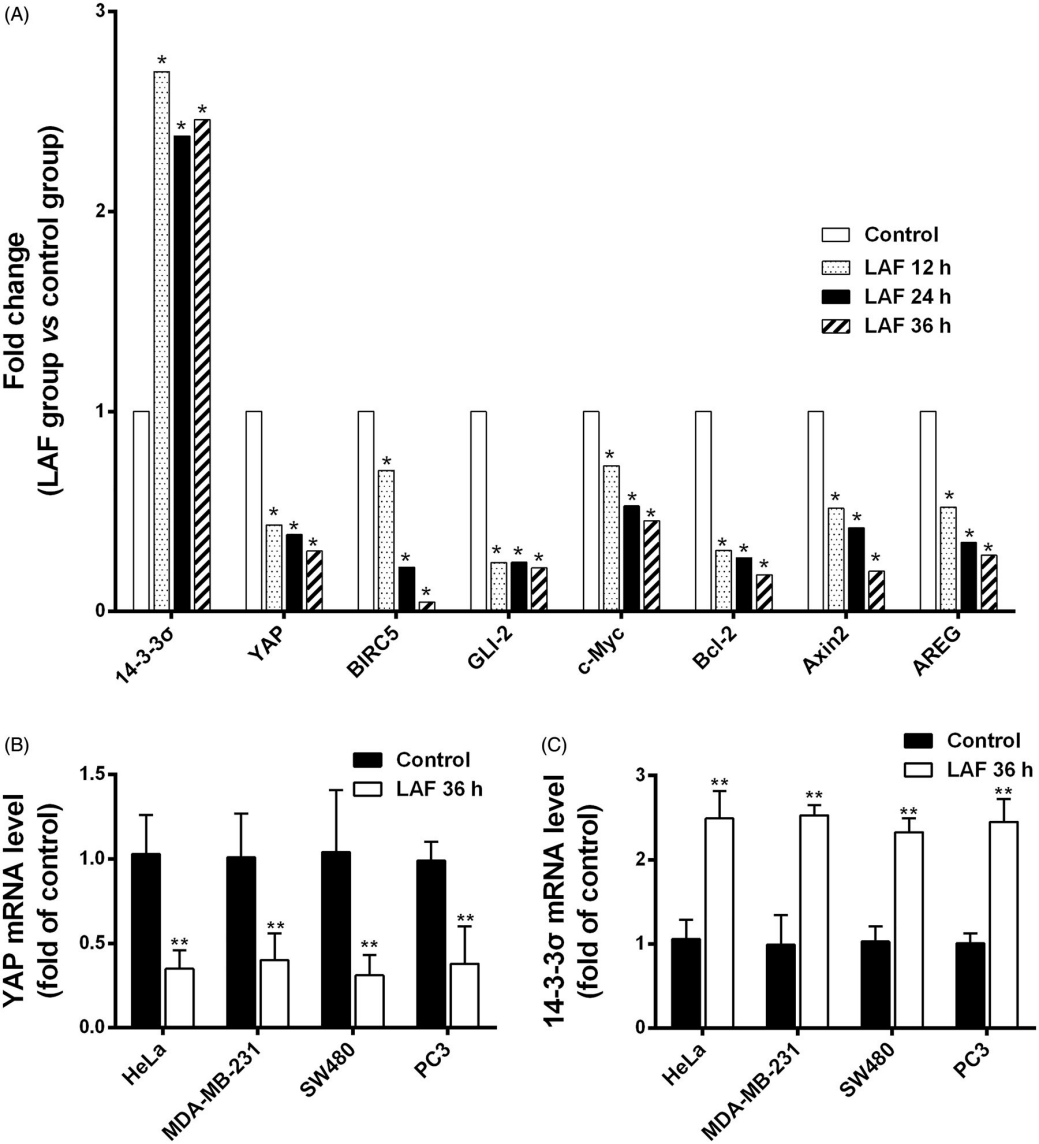
Patterns of mRNA level changes in the Hippo signalling pathway induced by Lappaol F (LAF). For transcriptome analysis, SW480 cells were treated with 50 µmol/L LAF for 12, 24 or 36 h. For quantitative RT-PCR, cancer cells (HeLa, MDA-MB-231, SW480 and PC3) were treated with 50 µmol/L LAF for 36 h. (A) Differentially expressed genes (fold change ≥ 2 and false discovery rate < 0.01) involved in the Hippo pathway. (B) YAP mRNA levels measured by quantitative RT-PCR. (C) 14-3-3σ mRNA levels measured by quantitative RT-PCR. All data are expressed as the mean ± SD (transcriptomic analysis, *n* = 3; quantitative RT-PCR, *n* = 6). **p < 0.01, significantly different from the control without LAF treatment. AREG: amphiregulin; Axin2: axis inhibition protein 2; Bcl-2: B cell lymphoma/leukemia-2; BIRC5: survivin/baculoviral IAP repeat containing 5; c-Myc: cellular myelocytomatosis oncogene; GLI2: glioma-associated oncogene family zinc finger 2; YAP: Yes-associated protein.

To identify the anticancer targets of LAF, transcriptome sequencing data from SW480 cells in the control and LAF treatment groups were analysed. The complete raw data files were deposited in the NCBI Sequence Read Archive (SRA) with the following link: https://www.ncbi.nlm.nih.gov/sra/PRJNA607632. The DEGs enriched in KEGG pathways were mainly involved in cancer pathways, cellular processes and environmental information processing such as the Hippo-YAP pathway. Interestingly, most of the DEGs in the Hippo signalling pathway were downstream genes, including YAP and its target genes. Compared to untreated cells, cells treated with LAF for 12, 24 or 36 h had strikingly lower transcriptional levels of YAP and its target genes, such as survivin/baculoviral IAP repeat-containing 5 (BIRC5), glioma-associated oncogene family zinc finger 2 (GLI2), cellular myelocytomatosis oncogene (c-Myc), B cell lymphoma/leukemia-2 (Bcl-2), axis inhibition protein 2 (Axin2) and amphiregulin (AREG), but markedly higher levels of 14-3-3σ, which is associated with cytoplasmic retention and degradation of YAP (Figure 3(A)). In addition, the results of quantitative RT-PCR also showed downregulation of YAP and an upregulation of 14-3-3σ after LAF treatment (Figure 3(B,C)).

### LAF decreased the protein levels, nuclear localisation and transcriptional activity of YAP

**Figure 4.**
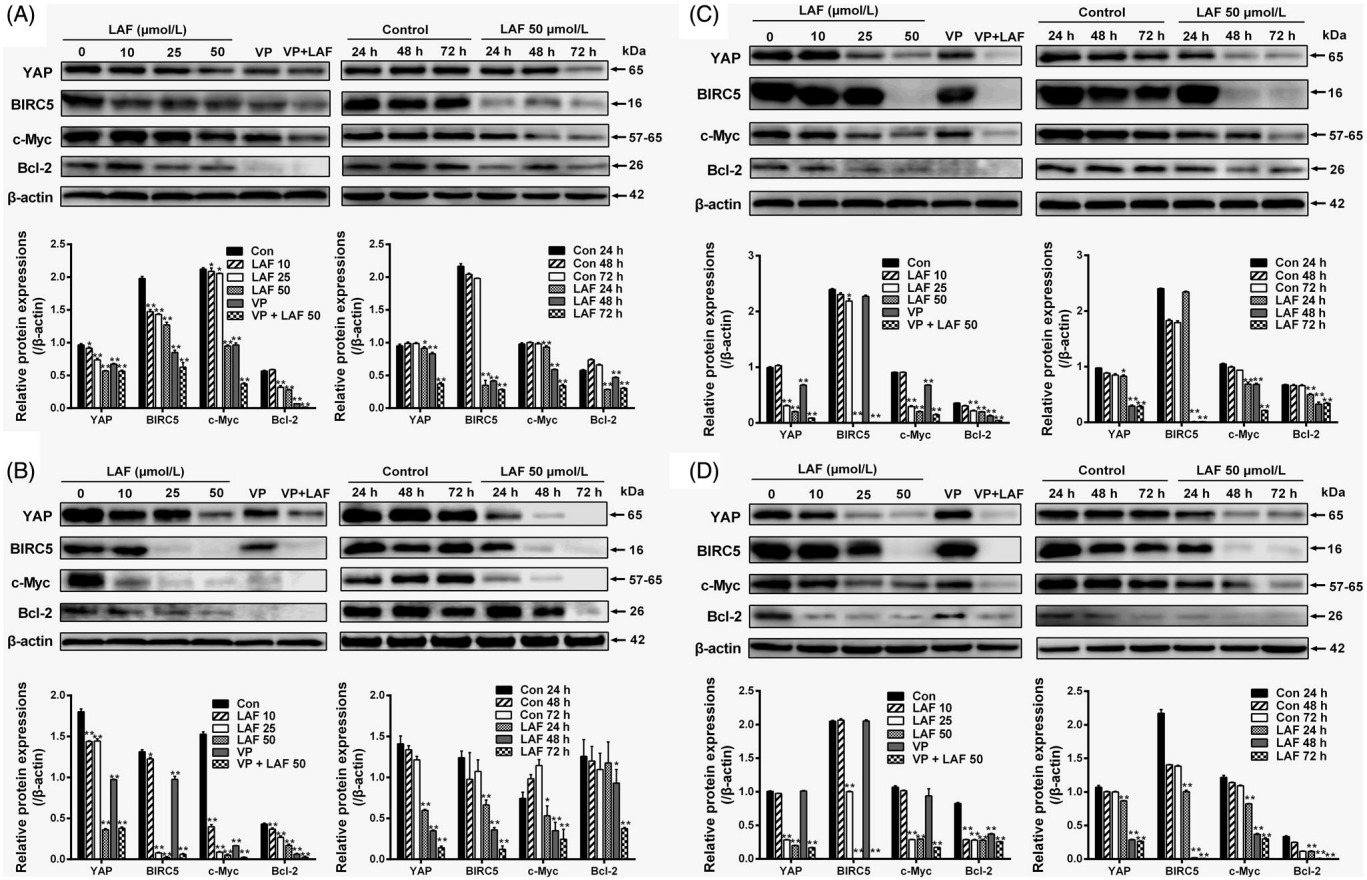
Lappaol F (LAF) downregulated the expression of YAP and its target genes in diverse cancer cell lines. The cells were exposed to LAF at different concentrations for 48 h (left side) or at 50 µmol/L LAF for different durations (right side). VP, a known YAP inhibitor, was added (1 µmol/L) as a positive control. Protein levels were determined by western blotting. (A) HeLa cells. (B) MDA-MB-231 cells. (C) SW480 cells. (D) PC3 cells. All data are expressed as the mean ± SD (*n* = 3). *p < 0.05, **p < 0.01, significantly different from the control without LAF treatment at the corresponding time points. Bcl-2: B cell lymphoma/leukemia-2; BIRC5: survivin/baculoviral IAP repeat containing 5; c-Myc: cellular myelocytomatosis oncogene; VP: verteporfin; YAP: Yes-associated protein; VP + LAF: LAF (50 µmol/L) combined with VP (1 µmol/L).

**Figure 5.**
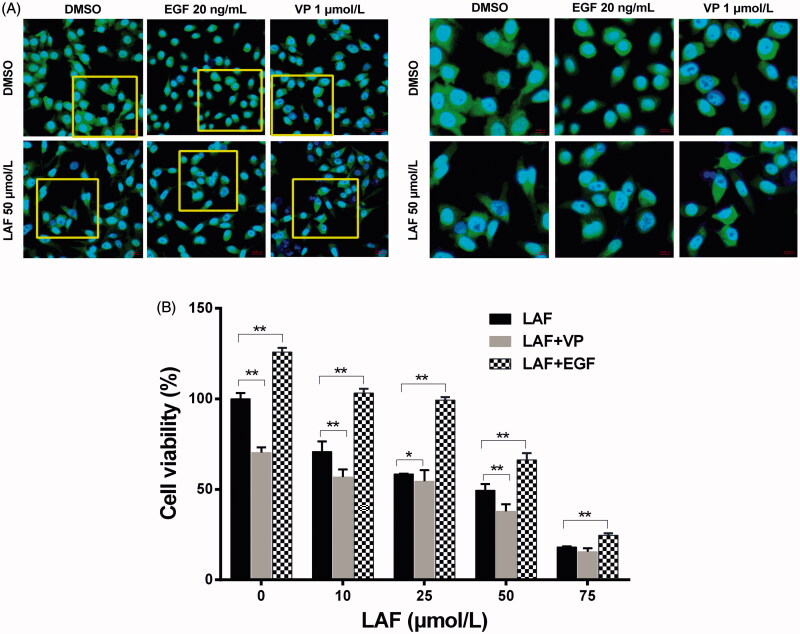
Lappaol F (LAF) decreased the nuclear accumulation of YAP. (A) Cellular location of YAP (green color) in HeLa cells treated with LAF (50 µmol/L), VP (1 µmol/L) or EGF (20 ng/mL) alone or simultaneously for 48 h. YAP was detected by immunofluorescence staining, while the nuclei (blue color) were detected by staining with 4,6-diamino-2-phenyl indole. Pictures at the right side (scale bars: 10,000 nm) are an enlargement of yellow frames in the pictures at the left side (scale bars: 20,000 nm). (B) Viability of HeLa cells treated with LAF (0, 10, 25, 50 or 75 µmol/L) alone or simultaneously with VP (1 µmol/L) or EGF (20 ng/mL) for 48 h measured by sulforhodamine B assay. All data are expressed as the mean ± SD (*n* = 6). *p < 0.05, **p < 0.01, significantly different from the control group. DMSO: dimethyl sulfoxide; EGF: epidermal growth factor; VP: verteporfin; YAP: Yes-associated protein.

Based on the above results, the protein levels and cellular localisation of YAP were verified by western blotting and immunofluorescence staining. In HeLa, MDA-MB-231, SW480 and PC3 cells, the total YAP level was remarkably downregulated by LAF (Figure 4). Further studies revealed that LAF reduced the nuclear accumulation of YAP in HeLa cells (Figure 5(A)). As a result of being excluded from the nucleus, the transcriptional activity of YAP decreased, leading to the downregulation of target genes, including BIRC5, c-Myc and Bcl-2 (Figure 4). Furthermore, LAF enhanced the inhibitory effect of VP but weakened the stimulatory effect of EGF on YAP (Figure 5(A)) as well as the proliferation of HeLa cells (Figure 5(B)).

### LAF inhibited YAP by upregulating 14-3-3σ

**Figure 6.**
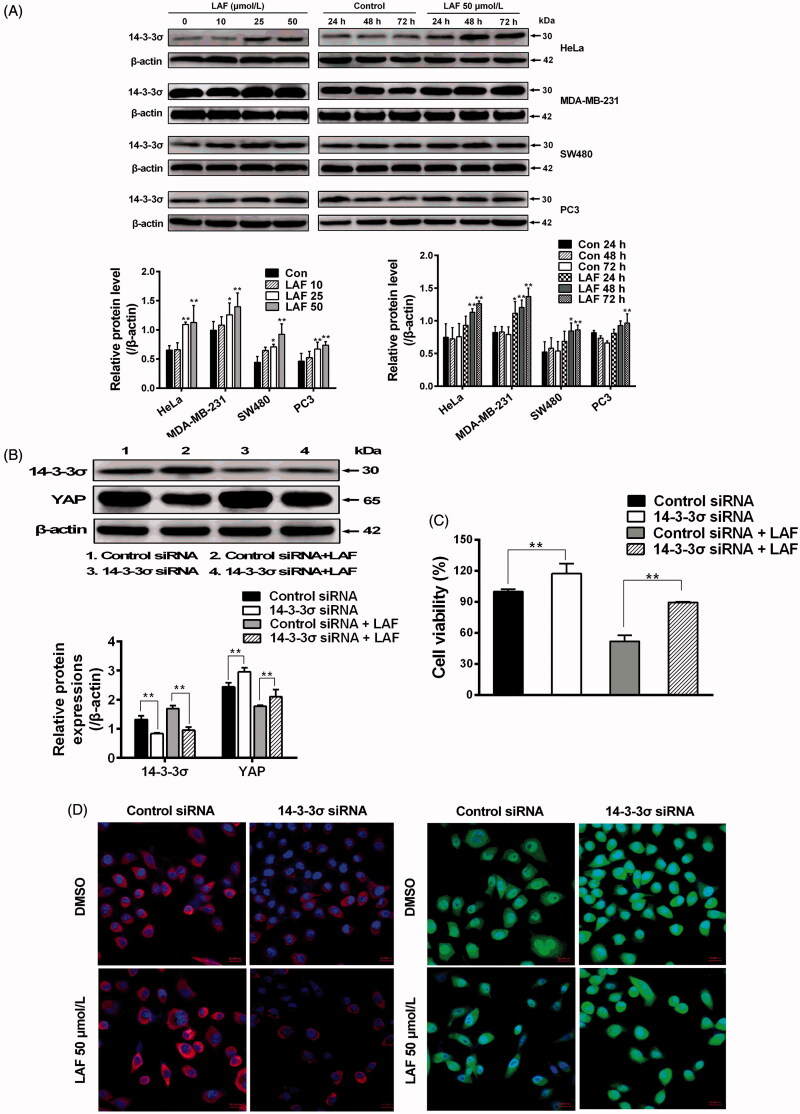
Lappaol F (LAF) inhibited YAP by increasing 14-3-3σ levels. (A) HeLa, MDA-MB-231, SW480 and PC3 cells were treated with LAF at different concentrations for 48 h (left side), or at 50 µmol/L LAF for different durations (right side). Western blotting was performed to evaluate the levels of 14-3-3σ. (B-D), HeLa cells were transfected with 14-3-3σ siRNA followed by LAF (50 µmol/L) treatment for 24 h. (B) The protein levels of 14-3-3σ and YAP were measured by western blotting. (C) Cell viabilities were measured by sulforhodamine B assay. (D) The expression and location of 14-3-3σ (red color) and YAP (green color) were observed by immunofluorescence staining. Nuclei (blue color) were stained with 4,6-diamino-2-phenyl indole. Scale bars represent 20,000 nm. All data are expressed as the mean ± SD (western blotting and immunofluorescence, *n* = 3; cell viability, *n* = 6). *p < 0.05, **p < 0.01, significantly different from the control siRNA group. DMSO: dimethyl sulfoxide; siRNA: small interfering RNA.

In contrast to YAP inhibition, western blotting showed that LAF promoted 14-3-3σ expression in HeLa, MDA-MB-231, SW480 and PC3 cells (Figure 6(A)). To confirm the effects of LAF on 14-3-3σ and YAP, siRNA technology was used to knockdown the expression of 14-3-3σ in HeLa cells. 14-3-3σ siRNA significantly decreased 14-3-3σ levels, resulting in an increase in YAP expression (Figure 6(B)), nuclear localisation (Figure 6(D)) and cell proliferation (Figure 6(C)). Moreover, after the use of 14-3-3σ siRNA, the inhibitory effect of LAF on YAP, including cell proliferation, was weakened (Figure 6(B,C,D)).

### LAF suppressed the growth of xenograft tumours *in vivo*

**Figure 7.**
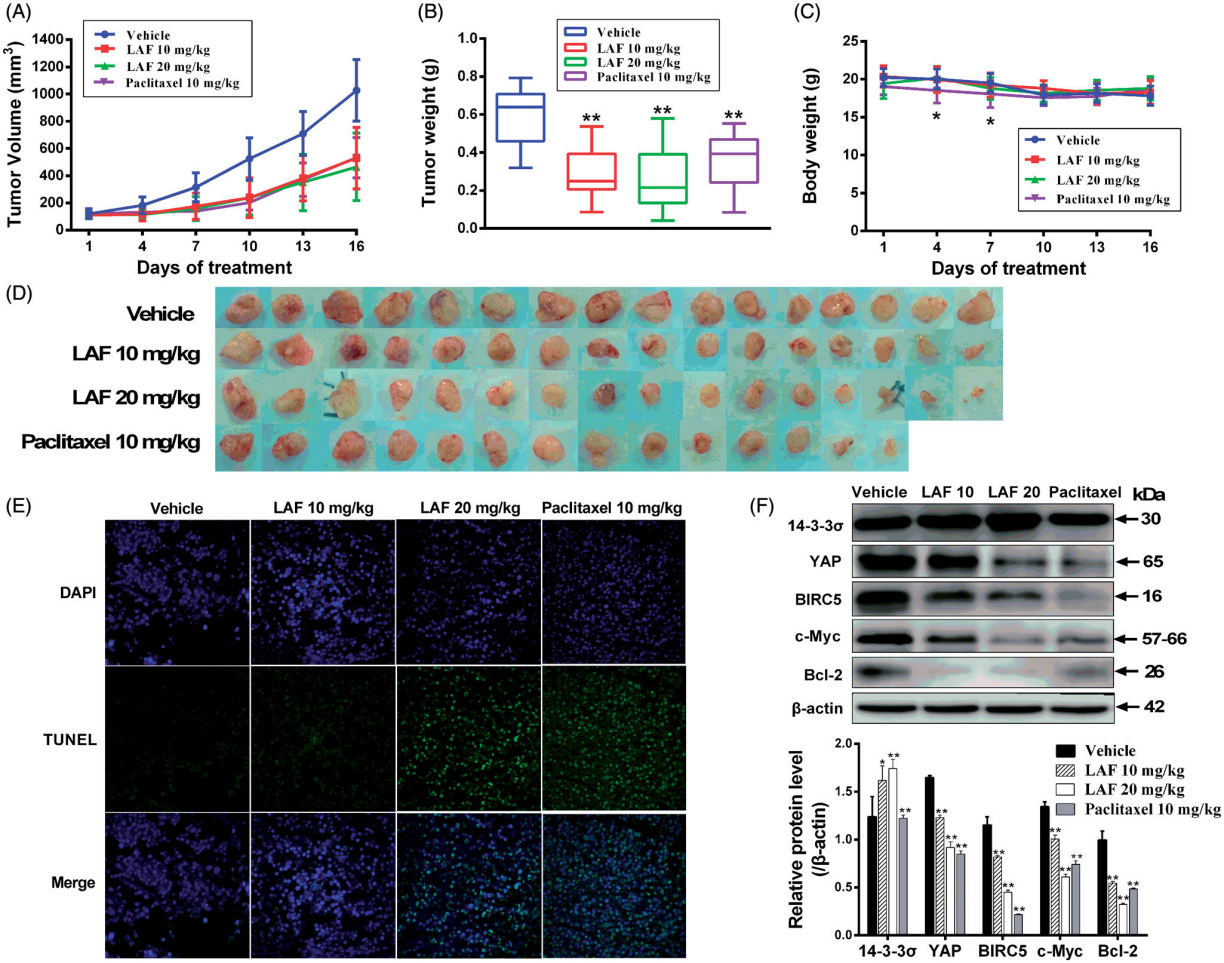
Lappaol F (LAF) suppressed the growth of tumour xenografts in nude mice. SW480 colon cancer cells were subcutaneously injected into the left and right back regions of nude mice to induce tumours. Subsequently, the tumour-bearing mice were intravenously administrated with LAF (*n* = 8) or vehicle (*n* = 8) for 15 consecutive days, or paclitaxel every other day (*n* = 7, one mouse in this group died on the 8th day of administration). (A) Tumour volumes. (B) Tumour weight. (C) Body weight. (D) Tumours. (E) The apoptosis of tumours was determined using TUNEL (magnification, × 400). (F) Expression levels of proteins in tumour tissues were detected by western blotting. All data are expressed as the mean ± SD. *p < 0.05, **p < 0.01, significantly different from the vehicle group. Bcl-2: B cell lymphoma/leukemia-2; BIRC5: survivin/baculoviral IAP repeat containing 5; c-Myc: cellular myelocytomatosis oncogene; DAPI: 4,6-diamino-2-phenyl indole; TUNEL: terminal deoxynucleotidyl transferase–mediated dUTP nick end labelling; YAP: Yes-associated protein.

We also investigated the effects of LAF on human colon cancer (SW480) xenografts in nude mice. Our results showed that continuous administration of LAF for 15 days significantly inhibited tumour growth (Figure 7(A,B,D)). Compared with the vehicle, LAF inhibited tumour size by 48% (10 mg/kg/d) and 55% (20 mg/kg/d) and reduced tumour weights by 52% (10 mg/kg/d) and 57% (20 mg/kg/d), without affecting the body weight of mice. The corresponding data in the paclitaxel group (10 mg/kg) were 48% and 40%, respectively. However, paclitaxel caused weight loss in mice on the 4th and 7th day of administration, and one death was recorded on the 8th day (Figure 7(C)). TUNEL assays demonstrated that LAF induced a significantly higher degree of apoptosis in tumour tissues (Figure 7(E)). Moreover, LAF dramatically upregulated the levels of 14-3-3σ in tumour tissues and downregulated the levels of YAP and its regulatory proteins, including BIRC5, c-Myc and Bcl-2 (Figure 7(F)).

## Discussion

In this study, we showed that LAF suppressed proliferation and induced apoptosis in various cancer cells. In addition, LAF strongly inhibited the growth of colon cancer xenografts in nude mice. We also found that the expression and nuclear localisation of YAP significantly decreased after LAF treatment. First, LAF downregulated YAP mRNA levels, indicating that LAF inhibited YAP at the transcriptional level. Furthermore, our results demonstrated that LAF increased 14-3-3σ at both the mRNA and protein levels. It has been reported that 14-3-3σ regulates cell proliferation by modulating cellular localisation and degradation of YAP (Sambandam et al. 2015; Wang et al. 2016). In the canonical Hippo signalling pathway, 14-3-3σ binds to the YAP pS127 site and prevents YAP from entering the nucleus (Schumacher et al. 2010). Subsequently, the 14-3-3-YAP complex trapped in the cytoplasm is ubiquitinated by the SCF^β-TRCP^ E3 ubiquitin ligase, resulting in the degradation of YAP (Zhao et al. 2010). Herein, the suppression of 14-3-3σ with siRNA resulted in an increase in YAP, suggesting that the expression of 14-3-3σ was negatively correlated with YAP. LAF increased 14-3-3σ levels, accompanied by a decrease in YAP expression and nuclear localisation. 14-3-3σ siRNA partially antagonised the effects of LAF on YAP, indicating that LAF inhibited YAP by upregulating 14-3-3σ, but was not completely dependent on 14-3-3σ. Therefore, LAF inhibited the expression and function of YAP through two pathways: transcriptional and 14-3-3σ-induced post-translational regulation.

Our previous results revealed that LAF induced cell cycle arrest, which was related to a decrease in cyclin B1 and CDK1 levels, and an increase in p21 and p27 (Sun et al. 2014). However, the upstream targets remain unclear. In this study, LAF inhibited YAP resulting in downregulation of YAP-driven gene expression, which ultimately led to proliferation inhibition and apoptosis promotion in human cancer cells. YAP, the final effector of the Hippo signalling pathway, plays a crucial role in cellular proliferation and apoptosis. Dysregulation of the Hippo pathway upstream leads to nuclear translocation of YAP, which activates the transcription of many growth-promoting and anti-apoptotic genes, including BIRC5, c-Myc and Bcl-2 (Zeng & Hong 2008; Chen et al. 2018). Moreover, several studies have reported that YAP stimulates cell cycle progression by upregulating cyclins and cyclin-dependent kinases (CDKs), and downregulating CDK inhibitors (Mizuno et al. 2012; Cao et al. 2014; Jang et al. 2017; Liu et al. 2017; Takeuchi et al. 2017). Therefore, our results suggest that LAF-induced suppression of cell proliferation may be due to YAP inactivation, leading to cell cycle arrest and apoptosis.

In addition to promoting tumour growth, YAP is believed to play a pivotal role in cancer metastasis (Lamar et al. 2012; Sharif & Wellstein 2015) and chemotherapy resistance (Zhao et al. 2014; Oku et al. 2015; Song et al. 2018). YAP overexpression has been observed in human cervix, breast, colon, prostate, liver, pancreas, lung and ovarian cancer, renal cell carcinoma, cholangiocarcinoma cells and tissue specimens, indicating a close relationship with cancer occurrence, development and metastasis (Zhao et al. 2007; Steinhardt et al. 2008; Wang et al. 2013; Cao et al. 2014; He et al. 2015; Kim et al. 2015; Pei et al. 2015; Li et al. 2017). Clinical studies have shown that YAP levels are negatively correlated with prognosis and survival rates in patients with colorectal, breast and ovarian cancer (Wang et al. 2013; Xia et al. 2014; Kim et al. 2015). Therefore, YAP is considered a potential anticancer drug target. In fact, some clinical anticancer drugs have been shown to have an indirect inhibitory effect on YAP. For example, paclitaxel increases YAP phosphorylation by activating CDK1 (Yang et al. 2013), dasatinib interferes with the binding of the YAP/β-catenin/TBX5 complex to Bcl-2L1 and BIRC5 promoters by inhibiting YES1 (Rosenbluh et al. 2012), and pazopanib inhibits YAP nuclear translocation by inhibiting vascular endothelial growth factor receptor and platelet-derived growth factor receptor signalling (Oku et al. 2015). Recently, VP, a photosensitizer used for the treatment of neovascular macular degeneration, was shown to suppress tumours by inhibiting YAP, independent of light activation (Brodowska et al. 2014). However, because of its low solubility and stability and YAP-independent effects, VP may not be a suitable drug for YAP-targeting in cancer treatment (Wu & Yang 2018). A further study revealed that VP promoted the expression of 14-3-3σ, leading to retention and degradation of YAP in the cytoplasm (Wang et al. 2016), similar to the mechanism by which LAF appears to inhibit YAP. Thus, LAF may be a promising candidate drug for cancer therapy, and its potential drug ability deserves further evaluation.

## Conclusions

The results of this study suggest that LAF inhibits tumours *in vitro* and *in vivo* by suppressing YAP through transcriptional and 14-3-3σ-induced post-translational regulation. This work will help to elucidate the pharmacological mechanism of the anticancer activity of LAF and provide experimental evidence for further drug development.
